# Trends in Medicaid Enrollment and Disenrollment During the Early Phase of the COVID-19 Pandemic in Wisconsin

**DOI:** 10.1001/jamahealthforum.2021.4752

**Published:** 2022-02-04

**Authors:** Laura Dague, Nicolás Badaracco, Thomas DeLeire, Justin Sydnor, Alyssa Shell Tilhou, Donna Friedsam

**Affiliations:** 1 Bush School of Government & Public Service, Texas A&M University, College Station; 2 Department of Economics, University of Wisconsin–Madison, Madison; 3 McCourt School of Public Policy, Georgetown University, Washington, DC; 4 Department of Risk and Insurance, University of Wisconsin–Madison, Madison; 5 Department of Family Medicine, Boston Medical Center, Boston University School of Medicine, Boston, Massachusetts; 6 Institute for Research on Poverty, University of Wisconsin–Madison, Madison

## Abstract

**Importance:**

After the federal public health emergency was declared in March 2020, states could qualify for increased federal Medicaid funding if they agreed to maintenance of eligibility (MOE) provisions, including a continuous coverage provision. The implications of MOE provisions for total Medicaid enrollment are unknown.

**Objective:**

To examine observed increases in Medicaid enrollment and identify the underlying roots of that growth during the first 7 months of the COVID-19 public health emergency in Wisconsin.

**Design, Setting, and Participants:**

This population-based cohort study compared changes in Wisconsin Medicaid enrollment from March through September 2020 with predicted changes based on previous enrollment patterns (January 2015-September 2019) and early pandemic employment shocks. The participants included enrollees in full-benefit Medicaid programs for nonelderly, nondisabled beneficiaries in Wisconsin from March through September 2020. Individuals were followed up monthly as they enrolled in, continued in, and disenrolled from Medicaid. Participants were considered to be newly enrolled if they enrolled in the program after being not enrolled for at least 1 month, and they were considered disenrolled if they left and were not reenrolled within the next month.

**Exposures:**

Continuous coverage provision beginning in March 2020; economic disruption from pandemic between first and second quarters of 2020.

**Main Outcomes and Measures:**

Actual vs predicted Medicaid enrollment, new enrollment, disenrollment, and reenrollment. Three models were created (Medicaid enrollment with no pandemic, Medicaid enrollment with pandemic economic circumstances, and longer Medicaid enrollment with a pandemic-induced recession), and a 95% prediction interval was used to express uncertainty in enrollment predictions.

**Results:**

The study estimated ongoing Medicaid enrollment in March 2020 for 792 777 enrollees (mean [SD] age, 20.6 [16.5] years; 431 054 [54.4%] women; 213 904 [27.0%] experiencing an employment shock) and compared that estimate with actual enrollment totals. Compared with a model of enrollment based on past data and incorporating the role of recent employment shocks, most ongoing excess enrollment was associated with MOE provisions rather than enrollment of newly eligible beneficiaries owing to employment shocks. After 7 months, overall enrollment had increased to 894 619, 11.1% higher than predicted (predicted enrollment 805 130; 95% prediction interval 767 991-843 086). Decomposing higher-than-predicted retention, most enrollment was among beneficiaries who, before the pandemic, likely would have disenrolled within 6 months, although a substantial fraction (30.4%) was from reduced short-term disenrollment.

**Conclusions and Relevance:**

In this cohort study, observed increases in Medicaid enrollment were largely associated with MOE rather than new enrollment after employment shocks. Expiration of MOE may leave many beneficiaries without insurance coverage.

## Key Points

**Question:**

Are increases in Medicaid enrollment during the COVID-19 pandemic associated more with maintenance of eligibility (MOE) policy or employment shocks?

**Findings:**

In this cohort study of 792 777 Wisconsin Medicaid enrollees, a 13.5% increase in overall enrollment vs predicted enrollment during the pandemic was largely associated with MOE rather than novel increases in enrollment owing to employment shocks. Most increased enrollment was among beneficiaries otherwise unlikely to remain enrolled 6 months later.

**Meaning:**

The findings suggest that expiration of MOE may leave many Medicaid beneficiaries without insurance coverage.

## Teaser

This cohort study examines whether maintenance of eligibility requirements or employment shocks are associated with increased Medicaid enrollment during the first 7 months of the COVID-19 pandemic in Wisconsin.

## Introduction

Since the federal declaration of the public health emergency related to the COVID-19 pandemic in the US in March 2020, enrollment in Medicaid has increased 16% nationally,^1^ an increase of more than 11 million individuals.^2^ Enrollment growth occurred in every state, ranging from 10% to 31%.^3^ Although new enrollment and disenrollment is a normal feature of Medicaid enrollment dynamics, the public health emergency brought a key change to Medicaid policy: maintenance of eligibility (MOE) provisions authorized under the Families First Coronavirus Recovery Act.^4^ Specifically, the act increased the federal share of Medicaid funding to states by 6.2% through the end of the month that the public health emergency expires, providing that states maintain continuous coverage for Medicaid beneficiaries—unless the beneficiary requests voluntary termination, is no longer a resident, or dies.^5^ All states participated. Consequently, since March 18, 2020, Medicaid members have not been subject to eligibility redetermination or disenrollment regardless of whether circumstances might normally have rendered them ineligible. Beneficiaries would normally be required to complete eligibility renewals, report changes in income and other circumstances, and otherwise respond to requests for eligibility-related information when the Medicaid agency identifies a need.

In this study, we used administrative data to examine increases in Medicaid enrollment and identify the underlying roots of that growth during the first 7 months of the public health emergency. We examined 2 channels: the MOE continuous coverage provisions during the public health emergency and the COVID-19 pandemic-related economic downturn. We assessed the degree to which these factors contributed to observed growth in Medicaid enrollment during the public health emergency.

Alongside MOE provisions enabling expanded enrollment, the early months of the pandemic brought substantial employment disruptions and expectations that many workers would lose employer-sponsored insurance and seek Medicaid or marketplace coverage.^6^ Although some decreases in employer-sponsored coverage have occurred,^7^ research to date has not shown a direct association between Medicaid enrollment and the unemployment rate during the public health emergency.^8,9,10^ Moreover, early data suggest that there are large enrollment increases in some safety-net programs but small increases in Medicaid or marketplace coverage.^11^ These findings contradict trends in Medicaid enrollment nationally^1,2^ and raise questions about the role of MOE provisions and employment shocks in changes in Medicaid enrollment.

Disentangling the factors behind enrollment has implications for state budgets and Medicaid administrative workflows, particularly when the enhanced federal Medicaid share ceases with the expiration of the public health emergency. A substantial number of current Medicaid enrollees may no longer be eligible when the public health emergency declaration expires. The Centers for Medicare & Medicaid Services is working with states to prepare for the “unwinding” of the public health emergency in 2022, focusing on redeterminations, transitions to marketplace plans, and avoidance of coverage losses.^12^ This process and the role of MOE can also inform future policy in states considering strategies to reduce disruptions in Medicaid coverage.^13^

## Methods

In this cohort study, we constructed an individual-level panel data set of all nonelderly, nondisabled Medicaid beneficiaries by month from January 2015 through September 2020 using administrative data from Wisconsin’s online eligibility and enrollment portal for public benefits. Wisconsin has a unique partial expansion Medicaid program that covers adults up to 100% of the federal poverty level.^14^ The data contain individual monthly level information on eligibility (including income, income sources, and household composition) along with demographic information including age, sex, educational level, race and ethnicity, and county of residence. Participant race and ethnicity are generally self-identified but occasionally may be reported by caseworkers; participants in this study identified as American Indian, Asian, Black, Hispanic, Pacific Islander, and White. Individuals were followed up monthly as they enrolled in, continued in, and disenrolled from Medicaid. We defined someone as newly enrolled if they enrolled in the program after being not enrolled for at least 1 month and as disenrolled if they left and were not reenrolled within the next month. This study was deemed exempt from review and informed consent by the University of Wisconsin’s Institutional Review Board (Common Rule, Category 5). The study followed the Strengthening the Reporting of Observational Studies in Epidemiology (STROBE) reporting guideline.

### Statistical Analysis

To assess how much of the increase in Medicaid enrollment was associated with MOE, we estimated what Medicaid enrollment would have been between March and September 2020 in a counterfactual scenario in which there was no MOE. We then ascertained whether increased enrollment was associated with reduced disenrollment and churn vs new enrollment. This assessment required estimating the rates of remaining enrolled for those enrolled as of March 2020, reenrollment for those who disenrolled, and new enrollment in the absence of MOE. Using data from individuals enrolled in Wisconsin Medicaid from 2015 through 2017, we estimated a model of enrollment in each month as the sum of people who remained continuously enrolled from a benchmark date, people who disenrolled since then and reenrolled, and new enrollees not observed at the benchmark date. We assessed how well estimates matched the observed data from 2018 and 2019 and then applied them to 2020.

Additional model details are in the eAppendix in the Supplement. We describe them here in brief. To adjust for changes in composition between our testing and prediction cohorts (those enrolled as of March 2018, 2019, and 2020) and our estimation cohort (those enrolled as of March 2017), we estimated a propensity score for each cohort relative to the 2017 cohort and implemented nearest-neighbor matching to create versions of the 2017 cohort that aligned with each of the 2018, 2019, and 2020 cohorts. We estimated counterfactual enrollment for each cohort in 5 steps. First, we estimated the probability of continued enrollment in each cohort by applying the nonparametric survival curve for each corresponding matched version of the 2017 cohort. Second, to account for reenrollment after disenrollment, we estimated the probability someone was reenrolled in each month in each cohort after a disenrollment (conditional on disenrollment) using a logit model in each matched cohort. Applying these probabilities yielded the number of individuals disenrolling each month expected to be reenrolled in each subsequent month. Third, to account for new enrollment, we regressed the number of new enrollees on each calendar month from 2015 through 2017 and created a monthly estimate for 2017 through 2020, a specification that accounts for strong seasonal enrollment patterns. Fourth, we applied estimated nonparametric survival functions to each month’s estimated new enrollees (much as we did for existing enrollees in step 1) to obtain the total number of ongoing newly enrolled beneficiaries. Fifth, we sum estimated the monthly continuing enrolled, reenrolled, and newly enrolled individuals to obtain total enrollment.

Model 1 yielded estimates of what Medicaid enrollment would have been without the COVID-19 pandemic under similar economic circumstances as previous years and allowed us to decompose enrollment into its components (continued enrollment, reenrollment, and newly enrolled).

To consider the economic circumstances of the pandemic vs MOE, we incorporated information about recent employment experiences of Medicaid enrollees and the elasticity of new enrollment with respect to new unemployment claims (model 2). We matched enrollment data to wage reports from the Wisconsin unemployment insurance reporting system, available from the first quarter of 2017 to the second quarter of 2020. We then made 2 changes to model 1. First, each step described above was estimated separately for those who did and did not experience an employment shock, measured as any member of the Medicaid case having a decrease in unemployment insurance earnings of 50% or more from 1 quarter to the next, from the first quarter to the second quarter (March enrollees) or at the time of their enrollment (new enrollees). Second, we estimated new enrollment as a function of new unemployment claims^15^ and calendar month using 2017 through 2019 data and used estimates from this regression to predict new enrollment during 2020.

We also simulated longer enrollment associated with the COVID-19 pandemic–induced recession (model 3) by eliminating disenrollments among those with a recent employment shock but otherwise following the model 2 procedure. This simulation assumed that all of those individuals currently or newly enrolled in Medicaid who were experiencing an employment shock during the early public health emergency would remain continuously enrolled.

In all 3 models, we used a 95% prediction interval (PI) to express uncertainty in the enrollment predictions. These simulated intervals incorporated estimation error and sampling error in the prediction and are further described in the Supplement.

eTables 1 through 6 and the eFigure in the Supplement present details on model estimation and performance in 2018 and 2019 (the placebo periods). Mean absolute percentage error is 1.08 for model 1 and 0.62 for model 2. Mean absolute deviation is 8525 for model 1 and 4925 for model 2. These metrics are another way to think about uncertainty in the model forecasts. All analyses were performed using Stata/MP, version 17 (StataCorp LLC), Excel 2016 (Microsoft), and The Decision Tools Suite @Risk, version 8.2 (Palisade).

## Results

The study estimated ongoing Medicaid enrollment in March 2020 for 792 777 enrollees (mean [SD] age, 20.6 [16.5] years; 431 054 [54.4%] women and 361 723 [45.6%] men) and compared that enrollment with actual enrollment totals. Enrollees in March 2020 self-identified (or were sometimes categorized by caseworkers) as American Indian (24 924 [3.1%]), Asian (32 868 [4.1%]), Black (164 715 [20.8%]), Hispanic (109 810 [13.9%]), Pacific Islander (1949 [0.2%]), and White (428 944 [54.1%]) (95 107 [12.0%] participants had missing data on race and ethnicity; participants could choose more than 1 race or ethnicity, so totals do not add to 100%). Table 1 summarizes other characteristics of Medicaid enrollees during the study period. The average enrollee in the March 2020 cohort was similar to enrollees in earlier years across almost all characteristics. The public health emergency coincided with a substantial shift in economic circumstances in the population: among those enrolled in March of each year, 213 904 (27.0%) experienced an employment shock from the first quarter to the second quarter in 2020, roughly twice the mean in past years.

**Table 1.** Characteristics of Enrolled Wisconsin Medicaid Cohorts, 2017–2020^a^

Characteristic	No. (%)			
	2017 (n = 803 659)	2018 (n = 796 162)	2019 (n = 786 095)	2020 (n = 792 777)
Age, mean (SD)	20.3 (16.6)	20.3 (16.6)	20.4 (16.6)	20.6 (16.5)
Sex				
Male	363 814 (45.3)	361 588 (45.4)	356 983 (45.4)	361 723 (45.6)
Female	439 845 (54.7)	434 574 (54.6)	429 112 (54.6)	431 054 (54.4)
Race and ethnicity^b^				
American Indian	25 334 (3.2)	25 082 (3.2)	24 597 (3.1)	24 924 (3.1)
Asian	33 745 (4.2)	33 235 (4.2)	32 632 (4.2)	32 868 (4.1)
Black	168 525 (21.0)	166 420 (20.9)	163 423 (20.8)	164 715 (20.8)
Hispanic	113 075 (14.1)	113 332 (14.2)	110 006 (14.0)	109 810 (13.9)
Pacific Islander	1916 (0.2)	1888 (0.2)	1842 (0.2)	1949 (0.2)
White	461 952 (57.5)	446 942 (56.1)	432 803 (55.1)	428 944 (54.1)
Missing race and ethnicity	63 195 (7.9)	73 304 (9.2)	83 623 (10.6)	95 107 (12.0)
Educational level^c^				
High school diploma or higher	188 915 (23.5)	187 680 (23.6)	186 542 (23.7)	188 903 (23.8)
Educational data missing	296 995 (37.0)	294 809 (37.0)	294 060 (37.4)	300 517 (37.9)
Income % of FPL, mean (SD)	56.1 (61.3)	58.1 (63.0)	59.5 (66.0)	58.4 (80.6)
Employment shock	104 965 (13.1)	108 805 (13.7)	107 358 (13.7)	213 904 (27.0)
Eligibility type				
Childless adult	149 104 (18.6)	151 613 (19.0)	151 274 (19.2)	157 199 (19.8)
Parents	148 464 (18.5)	141 714 (17.8)	133 123 (16.9)	129 147 (16.3)
Child	419 637 (52.2)	427 257 (53.7)	414 290 (52.7)	392 474 (49.5)
Pregnant	19 463 (2.4)	19 350 (2.4)	18 985 (2.4)	18 073 (2.3)
Other eligibility^d^	66 991 (8.3)	56 228 (7.1)	68 423 (8.7)	95 884 (12.1)

Abbreviation: FPL, federal poverty level.

^a^ Information was derived from Wisconsin administrative data. Demographic characteristics of the enrolled Wisconsin nonelderly, nondisabled Medicaid population are shown in March of each year.

^b^ Individuals may have reported more than 1 race or ethnicity, so totals may add to more than 100%.

^c^ An excluded category (no high school diploma) is not shown, so totals may add to less than 100%.

^d^ Other eligibility includes extensions, transitional eligibility, and youth exiting foster care.

**Figure 1.**
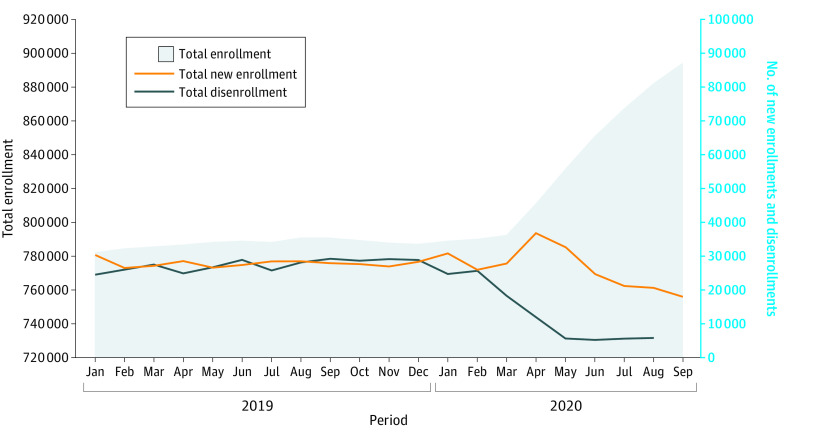
Trends in Wisconsin Medicaid Enrollment Information was derived from Wisconsin administrative data and shows monthly total enrollment (left axis) and new enrollment in and disenrollment from (right axis) Medicaid, where new enrollment and disenrollment implies at least 1 month out of the program. Disenrollment data for September 2020 were not available at the time of writing.

We analyzed trends in new enrollments, total enrollment, and disenrollments. Figure 1 shows that total enrollment across the state’s Medicaid programs had been steady at a mean of approximately 788 026 individuals per month in 2019. In April 2020, enrollment began to increase steadily until reaching 894 619 by September 2020, an increase of 13.5%.

Figure 1 shows that the increase was clearly not propelled by new enrollment overall. Relative to the previous mean of 28 026 new enrollees per month, new enrollment spiked briefly in April (31.7% increase) and May (16.9% increase) and then decreased 24.0% to a mean of approximately 21 297 per month from June through September 2020. A substantial decrease in disenrollments from 27 499 per month to 5659 per month (one-fifth of the previous level) appeared to be responsible for the increase.

**Figure 2.**
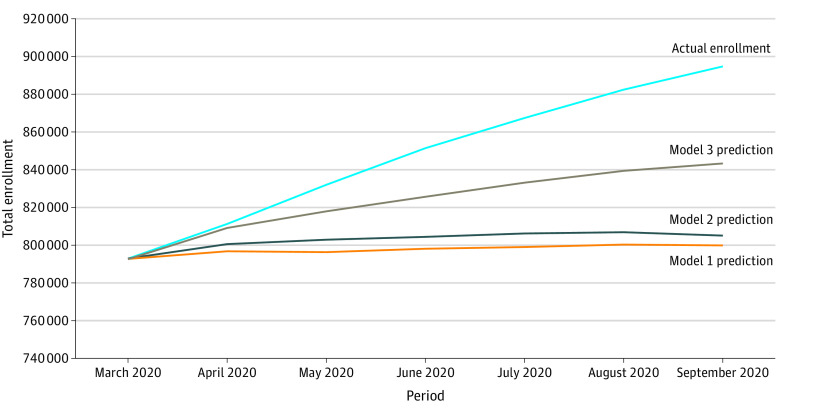
Predicted vs Actual Medicaid Enrollment Under Different Scenarios From March through September 2020 Information was derived from Wisconsin administrative data. Model 1 is based on only enrollment projections. Model 2 incorporates information on recent employment shocks. Model 3 uses model 2 estimates and simulates no disenrollment among those with a recent employment shock.

Figure 2 shows the model estimates compared with actual enrollment, summarized and decomposed by type of enrollment as described in Table 2. In model 1, based on past enrollment trends and demographic characteristics, actual total Medicaid enrollment by September 2020 (894 619) was 11.9% higher than the predicted 799 711 (95% PI, 795 782-803 677) enrollees (Table 2), which was then decomposed by source (ie, continuously enrolled since March 2020, temporarily disenrolled and reenrolled by September 2020, and newly enrolled after March 2020). The actual number of individuals with continuous enrollment since March 2020 (746 286) was 15.9% higher than the predicted 643 628 (95% PI, 642 895-644 361) enrollees. The number of ongoing newly enrolled individuals after March 2020 (139 281) was 19.5% higher than predicted at 116 574 (95% PI, 112 729-120 445) enrollees partly because the number of individuals (144 395) with any new Medicaid enrollment from April through September 2020 was 12.5% higher than the predicted 128 393 (95% PI, 124 210-132 593) individuals (eTable 6 in the Supplement). Reenrollment of enrollees who had disenrolled as of September 2020 (9052) was 77.1% lower than the 39 509 (95% PI, 39 117-39 899) enrollees estimated by the model. In addition, disenrollments of the March 2020 cohort who had not reenrolled by September were down 57.6% compared with the predicted estimate (46 491 vs 109 640 [95% PI, 108 810-110 474] individuals) (eTable 6 in the Supplement).

**Table 2.** Decomposition of Differences in Actual vs Predicted Wisconsin Medicaid Enrollment as of September 2020^a^

	No. of individuals (95% PI)			
	Continuously enrolled since March 2020	Temporarily disenrolled and reenrolled by September 2020	Newly enrolled after March 2020	Total September 2020 enrollment^b^
Actual enrollment^c^	746 286	9052	139 281	894 619
Model 1 predicted^d^	643 628 (642 895-644 361)	39 509 (39 117-39 899)	116 574 (112 729-120 445)	799 711 (795 782-803 677)
Difference, %	15.9	−77.1	19.5	11.9
Model 2 predicted^e^	640 880 (640 134-641 613)	41 104 (40 705-41 502)	123 146 (85 993-161 186)	805 130 (767 991-843 086)
Difference, %	16.4	−78.0	13.1	11.1
Model 3 predicted^f^	685 450 (684 836-686 059)	27 851 (27 535-28 169)	129 965 (90 055-170 812)	843 266 (803 334-884 215)
Difference, %	8.9	−67.5	7.2	6.1

Abbreviation: PI, prediction interval (incorporates estimation and sampling error).

^a^ Information was calculated from Wisconsin administrative data.

^b^ Sum of individuals continuously enrolled since March 2020, those temporarily disenrolled and reenrolled by September 2020, and those newly enrolled after March 2020.

^c^ Benchmark enrollment as of March 2020 was 792 777.

^d^ Model 1 is based only on enrollment projections.

^e^ Model 2 incorporates information on recent employment shocks.

^f^ Model 3 uses model 2 estimates and simulates no disenrollment among those with a recent employment shock.

Accounting for recent employment shocks modestly reduced the gap in predicted vs actual total enrollment (Figure 2). In model 2, total Medicaid enrollment was 11.1% higher by September 2020 (894 619) than the prediction of 805 130 (95% PI, 767 991-843 086) enrollees (Table 2). Continuous enrollment was up 16.4% (746 286 vs 640 880 enrollees [95% PI, 640 134-641 613 enrollees]), with the number of newly enrolled individuals (139 281) 13.1% higher than the predicted 123 146 (95% PI, 85 993-161 186) enrollees, and the actual number of reenrollees (9052) 78.0% lower than the predicted 41 104 (95% PI, 40 705-41 502) reenrollees. The number of individuals who disenrolled and did not reenroll decreased more than predicted by 58.0% (46 491 vs 110 793 [95% PI, 109 959-111 638] individuals) (eTable 6 in the Supplement). Cumulatively, total new enrollment was close to predicted (1.8%; 144 395 vs 141 879 [95% PI, 96 701-188 178] individuals) (eTable 6 in the Supplement). Of the total difference in predicted and actual enrollment, 18.0% (16 135 individuals) was explained by cumulative higher-than-expected new enrollment, whereas the remaining 82.0% represented a lack of disenrollment and reenrollment.

In model 3, which simulated disallowed disenrollment among those with recent employment shocks, the estimated difference between predicted and actual enrollment decreased to 6.1% (894 619 vs 843 266 individuals [95% PI, 803 334-884 215 individuals]) (Table 2). In other words, enrollment remained 6.1% higher than expected in the absence of MOE, assuming those without recent employment shocks had followed their typical enrollment cycles, those with recent employment shocks did not disenroll, and new enrollment was higher than typical because of increased employment shocks. This model reduced the gap between actual and counterfactual newly enrolled individuals to 7.2% (139 281 vs 129 965 individuals; 95% PI, 90 055-170 812 individuals) and slightly reduced the gap in reenrollees (9052) to 67.5% fewer than expected at 27 851 (95% PI, 27 535-28 169) individuals. The model 3 simulation also modestly reduced the gap in individuals who disenrolled and did not reenroll (41.5% lower; 46 491 actual vs 79 476 predicted individuals [95% PI, 78 786-80 158 individuals]) (eTable 6 in the Supplement).

Increased retention could be explained by reduced churning or by individuals who would be ineligible under non-MOE circumstances remaining enrolled. Although we cannot directly observe eligibility under non-MOE circumstances, short-term disenrollment followed by reenrollment is more likely to represent churning of eligible people, whereas longer-term disenrollment is more likely to reflect ineligibility. To assess the potential magnitude of these channels, we focused on the cohort originally enrolled in March 2020. We calculated the share of the gap in predicted vs actual retention of the March 2020 cohort (Table 2) coming from individuals predicted to be reenrolled in September 2020 vs those predicted to be no longer enrolled. In model 1, the gap in retention was 102 658 individuals, and temporary disenrollments with reenrollment were 30 457 lower than expected, suggesting 29.7% of the difference in predicted and actual retention came from individuals who would have left and quickly reenrolled. The remaining 70.3% (72 201 individuals) would not typically be enrolled 6 months later, 8.1% of the total enrolled caseload in September 2020. In model 2, the gap was similar at 105 406 with 32 052 fewer reenrollments than predicted and 73 354 more individuals who would not typically be enrolled 6 months later than predicted (8.2% of total September 2020 caseload). In model 3, the difference in predicted and actual retention was reduced to 60 836 individuals with 18 799 more reenrollments than expected, again approximately 30.9% of the gap. Because the absolute difference in predicted and actual enrollment was smaller than in models 1 and 2, this difference equaled 4.7% of the total September 2020 enrolled caseload. In summary, most excess retention of the initial cohort was explained by retention of individuals who would not typically be enrolled 6 months later, though a substantial fraction came from a reduction in churning.

## Discussion

This study assessed how observed increases in Medicaid enrollment reflect the retention of individuals under the continuous coverage provision of the MOE vs the enrollment of those newly eligible after economic displacement. We showed that, compared with a model of enrollment based on past enrollment data and incorporating the role of recent employment shocks, most ongoing excess enrollment was associated with the MOE rather than increases in enrollment associated with employment shocks. Furthermore, the analyses suggest that the continuous coverage provision may have promoted increased enrollment primarily via increased retention of those unlikely to remain otherwise enrolled 6 months later.

These findings highlight the difficult task of coverage redetermination as the public health emergency is expected to end in early 2022. The Centers for Medicare & Medicaid Services has issued 2 letters to state health officials with detailed instructions about how to prepare, possibly signaling concerns about precipitous coverage loss.^12,16^ The upcoming changes will affect state budgets, managed care entities, and provider organizations, all of which have come to rely on the higher federal matching funds tied to the increases in Medicaid enrollment.

Before the COVID-19 pandemic, Medicaid beneficiaries faced ongoing documentation requirements to maintain coverage. Such administrative burdens create potential coverage disruption even if the beneficiary remains eligible.^17^ We found that decreased churning explained nearly one-third of higher-than-predicted retention during the study period. Findings of the present study also showed that targeted policies can reduce disruptions and promote coverage continuity.

Results of this study are consistent with those of previous work showing that increased federal funds are not strongly correlated with changes in Medicaid enrollment nationally.^18^ The results are also consistent with findings that insurance coverage remained steady, unlike in previous recessions, with a larger increase in public coverage than decrease in employer-sponsored insurance^19^ and findings of weak correlation between Medicaid enrollment increases and unemployment rates.^7,8^ If enrollment increases operate largely through reduced churning, larger state programs with cumbersome enrollment processes before the COVID-19 pandemic might be expected to have the largest enrollment increases under MOE, and economic recovery may not be associated with a decrease in Medicaid enrollment.

### Limitations

This study has limitations. The COVID-19 pandemic is unprecedented, and the results of this study depend on the assumptions made and the data used for estimation. The nature of job loss may have changed throughout the pandemic in ways not captured. Medicaid enrollment may lag employment loss as unemployment benefits generally count as income for Medicaid eligibility. We estimate enrollment, not eligibility, so we cannot directly distinguish between reduced disenrollment and reenrollment owing to reduced administrative burden vs retained eligibility. In addition, our estimates may not be generalizable to other states.

## Conclusions

In this cohort study, we found that Medicaid enrollment in Wisconsin increased during the public health emergency more than expected based on previous enrollment patterns. The findings suggest that excess Medicaid enrollment could be largely attributed to MOE provisions rather than new eligibility tied to COVID-19 pandemic–related employment shocks. On expiration of the public health emergency, states face the sizeable task of transitioning a large fraction of their added caseload off of Medicaid. Without proper preparation, many current enrollees may face a period without insurance.

## Notes

Supplement. **eAppendix.** Additional Details for Models **eTable 1.** Margins From Propensity Score **eTable 2.** Postmatching Characteristics of 2017 Cohort **eTable 3.** Survival Probabilities **eTable 4.** Number of Entries, Exits, and Fraction of Original Cohort Reenrolled by Cohort **eTable 5.** Reenrollment Probabilities **eTable 6.** Total Exits and New Enrollees **eFigure.** Ratio of Predicted to Actual Enrollment by Model and Cohort-Month
