# Supplementary material for: Trends in Medicaid Enrollment and Disenrollment During the Early Phase of the COVID-19 Pandemic in Wisconsin
Source: JAMA Health Forum. 2022 Feb 4;3(2):e214752. doi: 10.1001/jamahealthforum.2021.4752 (PMC8903121; doi:10.1001/jamahealthforum.2021.4752)
Supplement: Supplement. — eAppendix. Additional Details for Models eTable 1. Margins From Propensity Score eTable 2. Postmatching Characteristics of 2017 Cohort eTable 3. Survival Probabilities eTable 4. Number of Entries, Exits, and Fraction of Original Cohort Reenrolled by Cohort eTable 5. Reenrollment Probabilities eTable 6. Total Exits and New Enrollees eFigure. Ratio of Predicted to Actual Enrollment by Model and Cohort-Month [file jamahealthforum-e214752-s001.pdf]

Supplementary Online Content

Dague L, Badaracco N, DeLeire T, Sydnor J, Tilhou AS, Friedsam D. Trends in Medicaid enrollment and disenrollment during the early phase of the COVID-19 pandemic in Wisconsin. *JAMA Health Forum*. 2022;3(2):e214752. doi:10.1001/jamahealthforum.2021.4752

eAppendix. Additional Details for Models

eTable 1. Margins From Propensity Score

eTable 2. Postmatching Characteristics of 2017 Cohort

eTable 3. Survival Probabilities

eTable 4. Number of Entries, Exits, and Fraction of Original Cohort Reenrolled by Cohort

eTable 5. Reenrollment Probabilities

eTable 6. Total Exits and New Enrollees

eFigure. Ratio of Predicted to Actual Enrollment by Model and Cohort-Month

This supplementary material has been provided by the authors to give readers additional information about their work.

© 2022 Dague L, et al. *JAMA Health Forum.*

eAppendix. Additional Details for Models

Additional Details for Model 1

As described in brief in the text, we estimate the model using data from 2015-2017 and predict enrollment from 2018-2020, using 2018 and 2019 as test periods.

To adjust for any differences in the composition of enrollees between our testing and prediction cohorts (those enrolled as of March 2018, 2019, and 2020) and our estimation cohort (those enrolled as of March 2017), we implement nearest neighbor propensity score matching to select samples from the 2017 cohort that is similar to the 2018, 2019, and 2020 cohorts. On the individual data, we estimate logit models with controls for gender, a quadratic in age, eligibility category (parent, child, pregnant woman, childless adult, or other), a quadratic in months of continuous enrollment, race (indicators for white, Black, Asian, Pacific Islander, American Indian, and missing information), Hispanic ethnicity, income, high school degree, citizenship, tribe member, and metropolitan county of residence that reflect the probability of being enrolled in March 2018, 2019, or 2020 relative to March 2017. The results of the logit model are presented in eTable 1. Each column 1 to 3 presents the probability of belonging to the sample in March 2018, 2019, and 2020, respectively, relative to March 2017. We then use these model estimates to construct the propensity score associated with being a member of the March 2018, 2019, and 2020 cohorts. We then select a nearest neighbor sample from the March 2017 cohort using these propensity scores to construct our estimation samples. Each year thus has a separate matched sample of March 2017 enrollees reflecting its demographics.

The first step in the model itself is estimating continued enrollment among those enrolled when the policy was in place (March 2020). We estimate the nonparametric (Kaplan-Meier) survival curve for individuals enrolled in March 2017 in each matched sample to generate nearest-neighbor propensity score matched versions of the survival curve for 2018-2020. This provides an estimate of the share of individuals initially enrolled in March that remained enrolled in subsequent months. These survival curves are listed in eTable 2 rounded to the nearest full percent. For example, the interpretation for someone enrolled in March 2018 would be: any individual enrolled in March 2018 has a probability of being enrolled in April 2018 of 97% and so on, based on estimates from the matching sample. Applying these probabilities to the number of enrollees in March of the corresponding year provides the number of enrollees that remain continuously enrolled.

Turnover, or churn, means that some individuals who exit in a month will re-enroll in subsequent months. Again using the matched sample of enrollees from the March 2017 cohort, we estimate the probability someone is from the March cohort is re-enrolled n months after an exit, with n=1, 2, …,12 (using a simple logit model and no additional controls). Using these probabilities we calculate the aggregate re-enrollment rates of the individuals that exit each month in each year and report these in eTable 3. For example, if a 2018 cohort individual exited at a given month, there is a 29% probability they will be enrolled again in Medicaid 2 months after the month they were not enrolled. We apply these probabilities to exiting enrollees each month.

Finally, to estimate new enrollment, we regress the number of new enrollees in each month (from April through February of the following year) on calendar month in 2015-2017 data. We then predict the average number of new enrollees by calendar month (e.g., average of Aprils in 2015, 2016, and 2017) as strong seasonal enrollment patterns make it important to control for time of year in some way. Since some of the new enrollees will also exit Medicaid, we apply survival functions to these new enrollees as well, estimated similarly as described above and presented in eTable 2. Survival functions for new enrollees tender to differ from the average cohort enrollee after the first six months but are otherwise very similar.

We then aggregate by month the number of predicted continued enrollees, new enrollees, and re-enrollees beginning with each March cohort in the test years (2018, 2019) and year of interest (2020) to come up with our total enrollment estimates.

© 2022 Dague L, et al. *JAMA Health Forum.*

Additional Details for Models 2-3

Models 2 and 3 augment Model 1 by incorporating information on the recent employment experience of new enrollees and continued enrollees. Survival curves and re-entry are estimated separately for those who have had a recent employment shock, defined as any member of the Medicaid case having a drop in UI wages of 50% or more from one quarter to the next, from Q1 to Q2 (March enrollees) or, for new enrollees, from the quarter prior to the quarter of their enrollment. We then predict total monthly new enrollment as a function of new unemployment claims and indicators for calendar month using 2017-2019 data from the Wisconsin Department of Workforce development (https://dwd.wisconsin.gov/uistats/). Because data on employment shocks are not observed for the second and third quarter of 2020 (the UI data are available only through Q2 2020), we need to make assumptions about the fraction of new enrollees with an employment shock on their case. We assume that it was 26% (as observed on average) through 2019 and remained elevated at 42% (as observed from Q1 to Q2 2020) throughout 2020.

Model Validation

eFigure 1 shows the fraction of enrollment predicted by the models for the six month time periods beginning April 2018, April 2019, and April 2020, where 1 means that the ratio of predicted to actual enrollment is one, or a perfect prediction. Throughout this period in 2018-2019, the models are generally off by a very small amount, with the largest absolute difference less than 3 percent. Model 1 tends to slightly overpredict total enrollment, less so in 2019 than in 2018, while Model 2 more closely matches actual enrollment in both years, slightly overpredicting in 2018 and slightly underpredicting in months 6-7 of 2019. The graph also shows the fraction of enrollment the models predict in 2020, which are immediately apparent as distinct from other years; even in month 2 when the usual margin of error is less than half a percent, Models 1-2 are already off by 1.8% and 1.2% respectively.

We also provide the Mean Absolute Percentage Error (MAPE) in addition to the Mean Absolute Deviation (MAD); both are standard forms of assessing the performance of a prediction model. The MAPE is 1.08 for Model 1 and 0.62 for Model 2. The MAD is 8,525 for Model 1 and 4,925 for Model 2.

As Model 3 is a simulation based on assumptions applied to the data only in the 2020 period using the inputs from Model 2, it has the same performance statistics as Model 2 in 2018-2019 and is excluded from eFigure1.

Method for generating prediction intervals:

We use a simulation approach to express the uncertainty in the enrollment predictions from the three models presented in Table 2 using 95% prediction intervals. These simulations incorporate two sources of uncertainty: estimation error and sampling error in the prediction. The estimation error arises because the estimates of the key model parameters, namely survival probabilities and re-enrollment probabilities, naturally contain the potential for error. The sampling error arises because even fixing a particular probability of survival or reenrollment for the population there will be randomness in how that survival/re-enrollment process plays out for the population.

To generate the prediction intervals on our estimates we create a simulation where each iteration re-calculates the projected enrollment numbers for each month (i.e., recreates the elements of Table 2). To incorporate the estimation error, the fixed survival probabilities and re-enrollment probabilities from our baseline estimates are each replaced with an independent random draw from a Normal Distribution with the mean set at the baseline probability estimate and the standard deviation set at the standard error on the mean estimated probability. In this way each of the probabilities applied in the model is allowed to vary in a range consistent with the estimated standard errors of that parameter. Then to account for sampling error, rather than simply multiplying those probability values by the starting sample sizes as we do in the baseline model, in the simulation we randomly draw enrollment numbers using the Binomial Distribution with the associate probability and sample size. Finally for the estimated number of newly enrolling members, we use the standard error on the predicted value from the regression model that generates those predictions and again draw independent random levels of new enrollment from a normal distribution with the mean set at the estimated level from the model and the standard deviation set to the standard error of the prediction model.

© 2022 Dague L, et al. *JAMA Health Forum.*

In each iteration the model draws random parameter levels for each monthly horizon, then draws an enrollment level from the associated binomial distribution. The final predictions are based on adding together these randomly drawn enrollment levels for different categories (e.g., continuing enrollment, re-enrolled members, etc...). We run the simulation for each of the models with 50,000 iterations. From those 50,000 iterations, we use the empirical 0.025 and 0.975 percentiles of the simulated enrollment numbers to establish our 95% prediction intervals for the model.

© 2022 Dague L, et al. *JAMA Health Forum.*

eTable 1. Margins From Propensity Score			
	(1)	(2)	(3)
VARIABLES	2018	2019	2020
Age	0.003***	0.005***	0.007***
	(0.000)	(0.000)	(0.000)
	<.001	<.001	<.001
Age Squared	-0.000***	-0.000***	-0.000***
	(0.000)	(0.000)	(0.000)
	<.001	<.001	<.001
Female	0.000	0.001	-0.001
	(0.001)	(0.001)	(0.001)
	0.610	0.215	0.497
American Indian	0.009***	0.015***	0.029***
	(0.003)	(0.003)	(0.003)
	0.008	<.001	<.001
Asian	0.009***	0.014***	0.032***
	(0.002)	(0.002)	(0.002)
	<.001	<.001	<.001
Black	0.011***	0.019***	0.036***
	(0.002)	(0.002)	(0.002)
	<.001	<.001	<.001
Hispanic	0.012***	0.017***	0.034***
	(0.002)	(0.002)	(0.002)
	<.001	<.001	<.001
Pacific Islander	0.002	0.004	0.021***
	(0.008)	(0.008)	(0.008)
	0.772	0.604	0.007
White	0.001	0.002	0.012***
	(0.002)	(0.002)	(0.002)
	0.548	0.189	<.001
Missing Race/Ethnicity	0.057***	0.109***	0.167***
	(0.002)	(0.002)	(0.002)
	<.001	<.001	<.001
FPL	0.000***	0.000***	0.000***
	(0.000)	(0.000)	(0.000)
	<.001	<.001	<.001
High School Degree	0.010***	0.017***	0.015***
	(0.001)	(0.001)	(0.001)
	<.001	<.001	<.001

© 2022 Dague L, et al. *JAMA Health Forum.*

	(1)	(2)	(3)
VARIABLES	2018	2019	2020
Education Missing	0.004***	0.013***	0.027***
	(0.001)	(0.001)	(0.001)
	<.001	<.001	<.001
Citizen	0.006**	0.009***	0.018***
	(0.003)	(0.003)	(0.003)
	0.030	0.002	<.001
Tribe Member	-0.001	-0.000	0.001
	(0.004)	(0.004)	(0.004)
	0.749	0.922	0.850
Residential County	-0.000***	-0.000***	0.000***
	(0.000)	(0.000)	(0.000)
	<.001	<.001	<.001
Months Enrolled	0.002***	0.002***	0.002***
	(0.000)	(0.000)	(0.000)
	<.001	<.001	<.001
Months Enrolled Squared	-0.000***	-0.000***	-0.000***
	(0.000)	(0.000)	(0.000)
	<.001	<.001	<.001
Parent	-0.022***	-0.041***	-0.061***
	(0.001)	(0.001)	(0.001)
	<.001	<.001	<.001
Child	0.021***	0.027***	0.000
	(0.002)	(0.002)	(0.002)
	<.001	<.001	0.840
Pregnant	-0.007**	-0.016***	-0.041***
	(0.003)	(0.003)	(0.003)
	0.021	<.001	<.001
Other Eligibility	-0.036***	0.021***	0.092***
	(0.002)	(0.002)	(0.002)
	<.001	<.001	<.001
Observations	1,599,363	1,588,541	1,595,316

Source: Authors' calculations from Wisconsin administrative data

Notes: Table shows marginal effects from logit model for propensity score. Includes coefficient, (standard error), and pvalue. *** p<0.01, ** p<0.05, * p<0.1

© 2022 Dague L, et al. *JAMA Health Forum.*

eTable 2. Postmatching Characteristics of 2017 Cohort

	2018 Match		2019 Match		2020 Match	
Age	20.3	(16.5)	20.4	(16.5)	20.7	(16.5)
Male	360,718	(45.3)	356,301	(45.4)	360,983	(45.6)
Female	435,166	(54.7)	428,761	(54.6)	430,854	(54.4)
American indian	24,548	(3.1)	24,063	(3.1)	24,754	(3.1)
Asian	32,763	(4.1)	31,651	(4.0)	32,232	(4.1)
Black	169,750	(21.3)	166,140	(21.2)	169,393	(21.4)
Hispanic	113,251	(14.2)	108,724	(13.8)	109,354	(13.8)
Pacific Islander	1,914	(0.2)	1,761	(0.2)	2,004	(0.3)
White	442,613	(55.6)	428,468	(54.6)	424,122	(53.6)
Missing Race/Ethnicity	74,092	(9.3)	85,833	(10.9)	95,017	(12.0)
HS degree	190,813	(24.0)	188,768	(24.0)	192,572	(24.3)
Education missing	289,640	(36.4)	290,940	(37.1)	296,668	(37.5)
Income % FPL	56.8	(62.0)	58.0	(63.6)	56.0	(64.0)
Eligibility Type						
Childless Adult	152,629	(19.2)	152,356	(19.4)	161,889	(20.4)
Parents	142,962	(18.0)	132,910	(16.9)	129,466	(16.4)
Child	426,630	(53.6)	412,229	(52.5)	385,463	(48.7)
Pregnant	18,891	(2.4)	18,959	(2.4)	18,084	(2.3)
Other Eligibility	54,772	(6.9)	68,608	(8.7)	96,935	(12.2)

Source: Authors' calculations from Wisconsin administrative data.

Notes: Table shows demographic characteristics of the matched 2017 cohorts.

© 2022 Dague L, et al. *JAMA Health Forum.*

eTable 3. Survival Probabilities

	March 2018	March 2019	March 2020	New Enrollees
	%	%	%	%
1	97%	97%	96%	97%
2	93%	93%	93%	94%
3	90%	90%	90%	90%
4	87%	87%	87%	87%
5	84%	84%	84%	84%
6	82%	82%	81%	80%
Unweighted Initial N	379,916	386,012	386,883	4,055,352

Source: Authors' calculations from Wisconsin administrative data

Notes: Table shows survival functions estimated on matched cohort and number at risk. New enrollees estimated on new enrollees 2015-2017.

© 2022 Dague L, et al. *JAMA Health Forum.*

eTable 4. Number of Entries, Exits, and Fraction of Original Cohort Reenrolled by Cohort

	2017		2018		2019		2020	
Employment Shock?	No	Yes	No	Yes	No	Yes	No	Yes
Entries								
April	25833	7911	23665	7828	21235	7499	20055	16856
May	15970	5206	15642	5170	15902	5304	17068	12952
June	16994	3677	16072	3726	16612	3909	14783	7682
July	13221	5229	13614	5571	14073	5839	19511*	
August	13357	4673	13256	4680	13658	5004	18989*	
September	12296	3068	11740	2905	13895	3627	16499*	
Exits								
April	27106	4816	25884	4934	23286	4477	13020	5245
May	23918	4210	23278	4433	19809	3958	7974	3276
June	24445	4264	22891	4074	20584	3935	3051	1354
July	21648	3801	22694	4223	21570	4033	2820	1205
August	22143	3725	21378	3881	18535	3299	3013	1224
September	21147	3604	21583	3911	19262	3493	2992	1317
Re-Enrolled								
May	5130	1065	4807	1237	4449	1106	1713	1030
June	11638	2372	11098	2581	9892	2272	3188	1813
July	17577	4915	17056	4751	15435	4348	6527*	
August	24447	6946	23820	6876	22048	6257	7845*	
September	31537	8681	30773	8592	29003	8056	9052*	

Source: Authors' calculations from Wisconsin administrative data

Notes: Table shows actual numbers of new entries (enrollees not in March cohort), exits (members of initial cohort who left), and re-enrolled (number of initial cohort who left and were re-enrolled) by employment shock category. *Employment shocks at entry and re-entry not observable after Q2 2020.

© 2022 Dague L, et al. *JAMA Health Forum.*

eTable 5. Reenrollment Probabilities

	P(Reentry|Exit)			
Months after	March 2018	March 2019	March 2020	
1	22%	22%	22%	
2	29%	29%	28%	
3	33%	32%	32%	
4	35%	35%	35%	
5	37%	37%	37%	

Source: Authors' calculations from Wisconsin administrative data

Notes: Table shows estimated probability a beneficiary is enrolled displayed number of months after exit

eTable 6. Total Exits and New Enrollees

	Exited, Not Re-enrolled by Sept. 2020	Total New Enrollees since March 2020
	(1)	(2)
Actual	46,491	144,395
Model 1 Predicted	109,640	128,393
	(108,810, 110,474)	(124,210, 132,593)
% difference	-57.6%	12.5%
Model 2 Predicted	110,793	141,879
	(109,959, 111,638)	(96,701, 188,178)
% difference	-58.0%	1.8%
Model 3 Predicted	79,476	141,879
	(78,786, 80,158)	(96,901, 188,163)
% difference	-41.5%	1.8%

Source: Authors' calculations from Wisconsin administrative data

Notes: Table shows number of benchmark cohort that exited and were not re-enrolled by September 2020 (column 1) and the sum of new enrollees not present in the March 2020 cohort from April-September 2020 (column 2). 95% confidence intervals in parantheses, details in text. 95% prediction errors that incorporate estimation and sampling error in parantheses.

© 2022 Dague L, et al. *JAMA Health Forum.*

© 2022 Dague L, et al. *JAMA Health Forum.*

eFigure. Ratio of Predicted to Actual Enrollment by Model and Cohort-Month

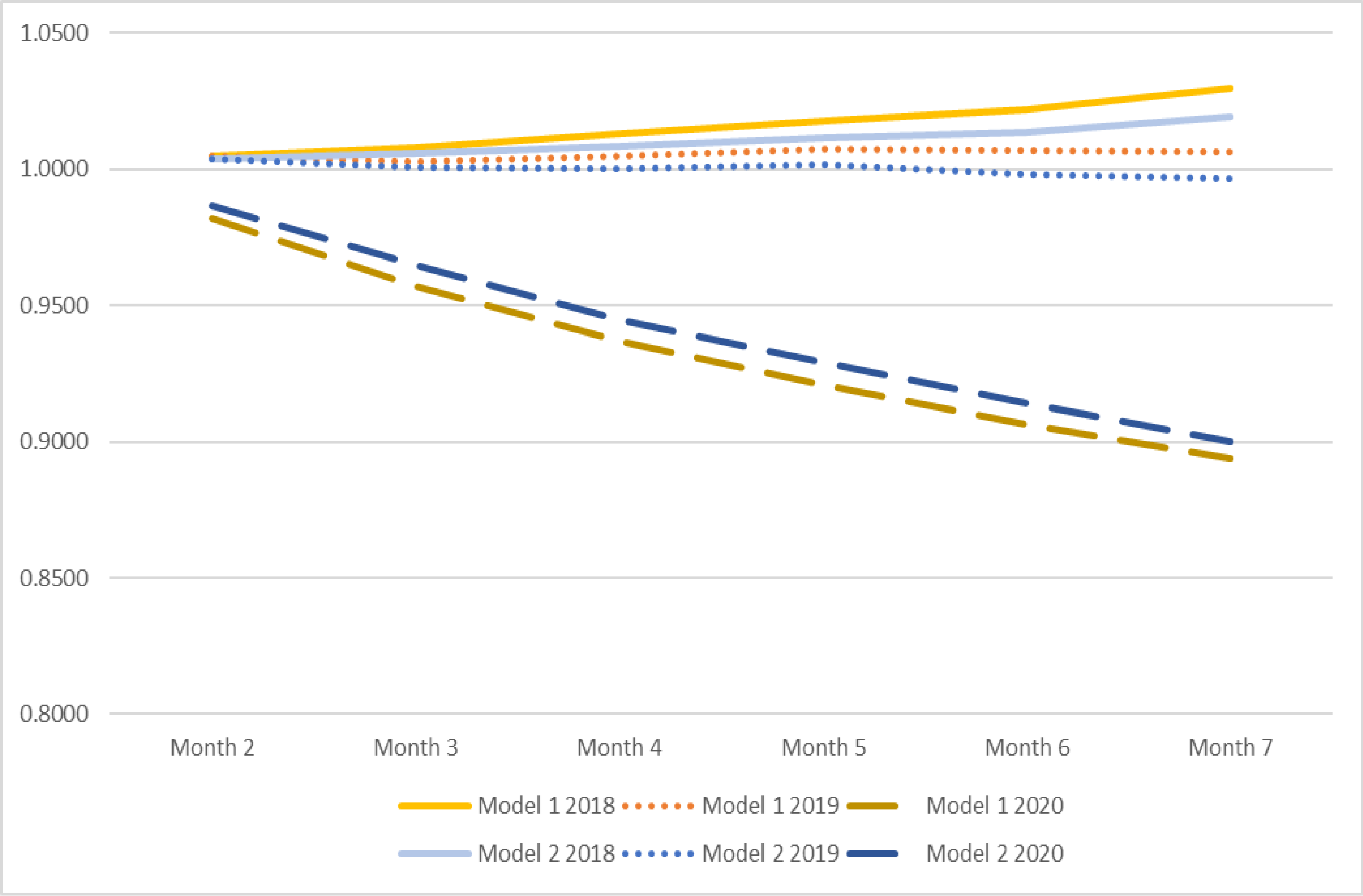

Source: Authors' calculations from Wisconsin administrative data
Notes: Figure shows ratio of model prediction to actual enrollment totals for test cohorts (2018, 2019) and for 2020 by month.
Model 1 is based only on enrollment projections. Model 2 incorporates information on recent employment shocks.

© 2022 Dague L, et al. *JAMA Health Forum.*
